# Loss of *Tsc1* in Osterix-expressing cells leads to greater bone mass and strength in mice

**DOI:** 10.1016/j.bone.2025.117695

**Published:** 2025-10-30

**Authors:** Han Kyoung Choi, Thomas Kim, Xiaoxi Wei, Karl J. Jepsen, Yuji Mishina, Nicholas Auyeung, Fei Liu

**Affiliations:** a Department of Biologic and Materials Sciences & Prosthodontics, University of Michigan School of Dentistry, Ann Arbor, MI, 48109, USA; b Department of Orthodontics, Jilin University School and Hospital of Stomatology, Changchun, Jilin, 130021, China; c Department of Orthopaedic Surgery and Biomedical Engineering, University of Michigan Medical School, Ann Arbor, MI, 48109, USA

**Keywords:** *Tsc1*, mTORC1, osteoblast, Osterix-Cre, bone mass, bone strength

## Abstract

Tuberous sclerosis complex 1 (*Tsc1*) negatively regulates mTORC1 signaling, but its role in postnatal skeletal development is not fully understood. Previous studies using various Cre drivers to target osteoblasts or mesenchymal lineage cells have generally shown higher bone mass accompanied by disorganized bone structure. However, our earlier study using Osx-Cre-mediated *Tsc1* deletion demonstrated that conditional knockout mice (CKO) had lower femoral trabecular bone at one month of age, but early lethality prevented later-stage assessment. Furthermore, how postnatal mTOCRC1 hyperactivation affects bone accrual and mechanical properties remains unknown. In this report, we first evaluated the cortical bone phenotype of one-month-old CKO mice using nanoCT, immunostaining, and quantitative PCR (qPCR). CKO mice exhibited greater cortical bone mass, elevated osteoblast markers (*Alpl, Bsp, Col1a1, Ocn*) and transcription factors (*Runx2, Osx*), enhanced periosteal proliferation in vivo, and upregulated proliferation of primary femur cortical bone-derived osteoblasts in vitro. To overcome early lethality and assess the impact of postnatal mTORC1 hyperactivation, we utilized the built-in doxycycline (Dox) Turn-Off system in Osx-Cre mice to suppress Cre activity until 2 months of age. Postnatal *Tsc1* deletion from 2 to 5 months led to robust cortical and trabecular bone gains in the femur, calvariae, and vertebrae. Picrosirius Red staining demonstrated that the femoral cortical bone in CKO mice exhibited organized collagen with lamellar features, indicating preserved tissue quality. Importantly, four-point bending tests demonstrated significantly improved femoral mechanical strength in CKO mice. In summary, our data reveal differential effects of *Tsc1* deletion on trabecular and cortical bone at an early postnatal stage, and show that postnatal deletion results in robust bone gain with enhanced mechanical strength. These findings provide a more complete understanding of *Tsc1*-mTORC1 signaling as a key regulator of bone mass and challenge the assumption that mTORC1 hyperactivation yields mechanically inferior bone.

## 1. Introduction

The tuberous sclerosis (TS) is a multisystem genetic disorder caused by inactivating mutations in either *TSC1* or *TSC2*, which encode hamartin and tuberin, respectively—key negative regulators of mechanistic target of rapamycin complex 1 (mTORC1) signaling [1]. Loss of either gene results in constitutive mTORC1 activation, leading to abnormal cell growth and proliferation in multiple tissues. Clinically, patients with TS present with benign tumors in various organs and characteristic skin lesions, along with skeletal abnormalities such as sclerotic bone lesions in the axial and craniofacial skeleton, fibrous dysplasia-like changes, jaw bone cysts, and dental enamel pitting [2–5]. These skeletal manifestations underscore the importance of mTORC1 signaling in bone biology and provide a clinical context for studying *Tsc1* deletion in osteoblast-lineage cells. Due to its prevalence, the sclerotic bone lesions have been included as a minor clinical diagnostic criterion in the international TS diagnostic criteria [6].

Genetic deletion of *Tsc1* or *Tsc2* in mice produces distinct skeletal phenotypes depending on the targeted cell population and timing of deletion. In neural crest–derived cells, *Tsc1* deletion by P0-Cre reproduces sclerotic craniofacial lesions through osteoprogenitor hyperproliferation [7]. In mesenchymal progenitors, *Tsc1* deletion by Prx1-Cre leads to a greater bone width and mass but impairs differentiation and mineralization in association with reduced bone quality, while suppressing osteoclast activity [8]. In committed osteoblast-lineage cells, *Tsc2* deletion by Ocn-Cre disrupts bone organization and systemic metabolism [9], and in Osterix-expressiong cells, *Tsc1* deletion by Osx-Cre has been reported to cause woven bone resulting from excessive proliferation and impaired maturation [10]. Our earlier study using Osx-Cre–mediated *Tsc1* deletion showed early lethality, reduced trabecular bone at one month, and decreased proliferation and differentiation of bone marrow–derived osteoblasts [11]. In mature osteoblasts and osteocytes, *Tsc1* deletion by 10 kb Dmp1-Cre reduces sclerostin, enhances bone formation, and causes osteosclerosis [12]. Together, these studies underscore that *Tsc1* deletion in the osteoblast lineage can lead to diverse outcomes, highlighting the need to clarify its effects on postnatal bone development and mechanical function.

Building on these prior findings, the present study aimed to further define the skeletal consequences of *Tsc1* deletion in Osx-expressing cells, with particular focus on postnatal bone development and mechanical function. Osx-Cre with a doxycycline-controlled system allowed us to either constitutively deleting *Tsc1* or delay deletion postnatally, enabling us to assess both early and later-stage skeletal phenotypes. Importantly, we directly measured bone mechanical properties—an aspect not previously examined in this genetic context.

## 2. Materials and methods

### 2.1. Mice

The generation of floxed *Tsc1*^F/F^ mice [13] and Osterix-Cre (Osx-Cre) mice [14] has been described previously. All mice were maintained on a C57BL/6 background after at least eight generations of backcrossing. For constitutive conditional knockout experiments, *Tsc1*^F/F^ mice were bred with Osx-Cre/+ mice to generate *Tsc1*^F/F^;Osx-Cre/+ (CKO) and littermate control mice, both mating pairs and offsprings were fed with regular diet without doxycycline. For postnatal deletion studies, the same breeding strategy was used except that breeding pairs were maintained on a doxycycline-containing diet (200 mg/kg) during mating and pregnancy. Offspring were kept with dams on the doxycycline diet until weaning and then maintained on the same diet until 2 months of age, after which they were switched to a regular diet to allow Cre activation for the next 3 months. Mice were housed under specific pathogen-free conditions at 22 ± 2 °C on a 12:12-h light/dark cycle with ad libitum access to food and water. All procedures were conducted in compliance with institutional and federal regulations and approved by the Institutional Animal Care and Use Committee at the University of Michigan. Investigators performing bone analyses were blinded to genotypes.

### 2.2. Nano-computed tomography (nanoCT) scanning and analysis

For femurs and the third lumbar vertebra, specimens were immobilized inside a plastic tube, and scanned using a Phoenix Nanotom M nanoCT (Waygate Technologies, Baker Hughes Company, Skaneateles, NY, USA). The X-ray tube was powered to 80 kV and 400 μA, utilized a diamond coated tungsten target, a 0.381 mm Aluminum filter, and was set to a spot size of 0. Imaging was done at 8 μm voxel size using an exposure time of 500 ms; 3 frames averaged and 1 skipped for each exposure. The sample stage rotated through 360 degrees and collected 2000 images per scan. Reconstruction of raw data was performed using Datos.rec version 2.6.1 (GE Inspection Technologies, LP, Skaneateles, NY, USA). For calvariae (skull), imaging was done at 12 μm voxel size and 1500 images were collected per scan. CT analyses were performed following previously established protocols for femur [15–17], calvariae [18–20], and vertebrae [17]. For femoral trabecular analysis, the distal femoral region of interest (ROI) was defined beginning at the junction of the growth plate and metaphyseal trabeculae, extending proximally for 10 % of the total femoral length. The ROI was contoured in the trans-verse plane using a splining algorithm (MicroView v2.2, GE Healthcare Pre-Clinical Imaging). Femoral cortical parameters were measured from a diaphyseal ROI encompassing 18 % of the bone length centered at the midshaft. For vertebral trabecular measurements, the ROI in the LC3 vertebra was isolated from the surrounding cortical shell by outlining serial areal contours at several levels and interpolating to construct a 3D trabecular volume. Vertebral cortical bone area was evaluated from a single slice positioned 0.63 mm below the cranial growth plate as described before [17]. For calvarial analysis, the frontal ROI (0.5 mm × 0.5 mm) was positioned 1.5 mm anterior to the coronal–sagittal suture junction and 1 mm lateral to the posterior frontal suture. The parietal ROI of the same dimensions was placed 1.5 mm posterior to the coronal–sagittal junction and 1.5 mm lateral to the sagittal suture. In both locations, the full bone thickness was included in the measurement.

### 2.3. RNA extraction and qRT-PCR

RNA extraction and qRT-PCR analyses were performed as described previously [15]. Total RNA was extracted using Trizol reagent (Invitrogen) following the manufacturer's protocol. Equivalent quantities of RNA were converted to cDNA with the SuperScript III First-Strand Synthesis Kit (Invitrogen) using oligo(dT) primers. The resulting cDNA was analyzed by quantitative RT-PCR with SYBR Green PCR Core re-agents (Qiagen, Valencia, CA, USA) using primers specific for *Alpl* (alkaline phosphatase), *Bsp* (bone sialoprotein), *Col1a1* (Collagen type I A1), *Runx2*, *Ocn* (osteocalcin), *Osx* (osterix), and 18S rRNA. The reported relative expression of each gene was normalized to 18S rRNA. Primer sequences were identical to those reported in our earlier studies [7,11,15,19].

### 2.4. Femoral osteoblasts culture

Primary osteoblasts were obtained from femoral cortical bone of 1 month-old control or *Tsc1* CKO mice. In brief, after flushing out the bone marrow and cutting out the trabecular bone area, cortical bone was chapped approximately 1–2 mm^2^ using scissors. After washing with PBS, cortical bone pieces of femurs were subjected to digestions. Two digestions were performed in an enzyme solution containing 1 mg/mL collagenase A (#11088793001, Roche) and 1.5 mg/mL Dispase II (#4942078001, Roche) at 37 °C on a 250 rpm shaking platform. After the digestion, bone chips were washed with α-MEM containing 10 % FBS and plated in the dishes with α-MEM containing 10 % FBS. Medium was changed 24 h later. Six days later, the osteoblasts that migrated out from bone chips were trypsinized and harvested. After centrifugation for 5 min at 1500 *g*, cell pellets were re-suspended in medium and plated in 12-well plates at a density of 3 × 10^4^ cells per well in α-MEM containing 10 % FBS for osteoblast differentiation experiments.

### 2.5. Osteoblast differentiation assay

Primary osteoblasts were seeded at 1 × 10^5^ or 3 × 10^4^ cells/well in 12-well plates. Osteogenic differentiation was induced and assessed as previously described [19]. The osteogenic medium containing 50mg/mL of ascorbic acid and 4 mM of beta glycerophosphate. The alkaline phosphatase (ALP) staining and Alizarin red (AR) staining were performed on day 7 and day 21, respectively.

### 2.6. Proliferation assay

Primary osteoblasts were seeded at 1 × 10^5^ cells/well in 12-well plates in osteogenic medium containing 50mg/mL of ascorbic acid and 4 mM of beta glycerophosphate. Cells were harvested on day 3 or day 7 and counted. Parallel cultures were fixed with 4 % paraformaldehyde and stained for Ki67 (Cell Signaling Technology, Beverly, MA, USA) as described previously [7,16,19].

### 2.7. Immunostaining

Paraffin sections were processed for immunostaining as described previously [7,16]. Paraffin sections were deparaffinized, rehydrated, and subjected to antigen retrieval. For immunohistochemistry, endogenous peroxidase activity was quenched with 0.3 % H_2_O_2_ in methanol (30 min), followed by overnight incubation at 4 °C with anti-Osterix antibody (Abcam, ab22552, USA). Signal was visualized using a horse-radish peroxidase–streptavidin system (Dako) and counterstained with hematoxylin (Sigma-Aldrich). For immunofluorescence, sections were incubated overnight at 4 °C with anti-Ki67 antibody (Spring, M306), washed, and then exposed to Alexa Fluor–conjugated secondary antibody (Life Technologies, A-21206) for 1 h at room temperature. After washing, slides were mounted with ProLong^®^ Gold Antifade with DAPI. Positive cells and bone perimeter were quantified in ImageJ using multipoint and segmented line tools.

### 2.8. Western blot

Western blot was performed as previously described [7,16,21]. Antibody information is available in earlier publications [7,11].

### 2.9. Four-point bending tests

Intact femurs were loaded to failure in 4-point bending at 0.05 mm/s using a servohydraulic materials testing system (MTS 858 MiniBionix, Eden Prairie, MN, USA). Femurs were kept moist with PBS and loaded at room temperature with the anterior side subjected to tensile loads. Stiffness (S), maximum load (ML), post-yield deflection (PYD), and work-to-fracture (Work) were calculated from the load-deflection curves, as described previously [22].

### 2.10. Histology and picrosirius red staining

Femora from 5-month-old male control and CKO mice were fixed in 4 % paraformaldehyde and decalcified in 14 % EDTA (pH 7.4) at 4 °C. Bones were paraffin-embedded, sectioned at 6 μm, and processed for hematoxylin and eosin (H&E) staining to evaluate morphology. For collagen assessment, adjacent sections were incubated in 0.1 % Sirius Red (Direct Red 80, Sigma) prepared in saturated picric acid, washed with 0.5 % acetic acid, dehydrated through graded alcohols, cleared in xylene, and coverslipped. H&E sections were imaged under bright-field microscopy, while Sirius Red–stained sections were examined under polarized light with identical exposure settings across groups. Collagen organization was judged qualitatively: red–orange birefringence was interpreted as aligned, lamellar collagen, whereas green birefringence was indicative of less mature or loosely organized fibers.

### 2.11. Statistical analysis

Comparisons between two groups were made using Student's *t*-test. For experiments with more than two groups, one-way ANOVA followed by Tukey's post hoc test was used. Statistical analyses were performed using GraphPad Prism version 7.0, and *p* < 0.05 was considered statistically significant.

## 3. Results

### 3.1. Tsc1 deletion in Osterix-expressing cells in embryos leads to higher femoral cortical bone and calvarial bone mass in one-month-old mice

To exam the role of *Tsc1* in early osteoblast lineage cells, we used Osterix-Cre (Osx-Cre) [14] to generate *Tsc1* conditional knockout (CKO, *Tsc1*^F/F^;Osx-Cre) mice, along with littermate controls: floxed *Tsc1* (*Tsc1*^F/F^), Osx-Cre/+ (Osx-Cre), and *Tsc1*^F/+^;Osx-Cre mice (conditional heterozygous, Chet). Using this mouse model, our previous work focused on the trabecular bone compartment [11]. Here, we performed nanoCT analysis to assess cortical bone parameters in the femur and bone mass in the calvaria.

**Fig. 1.**
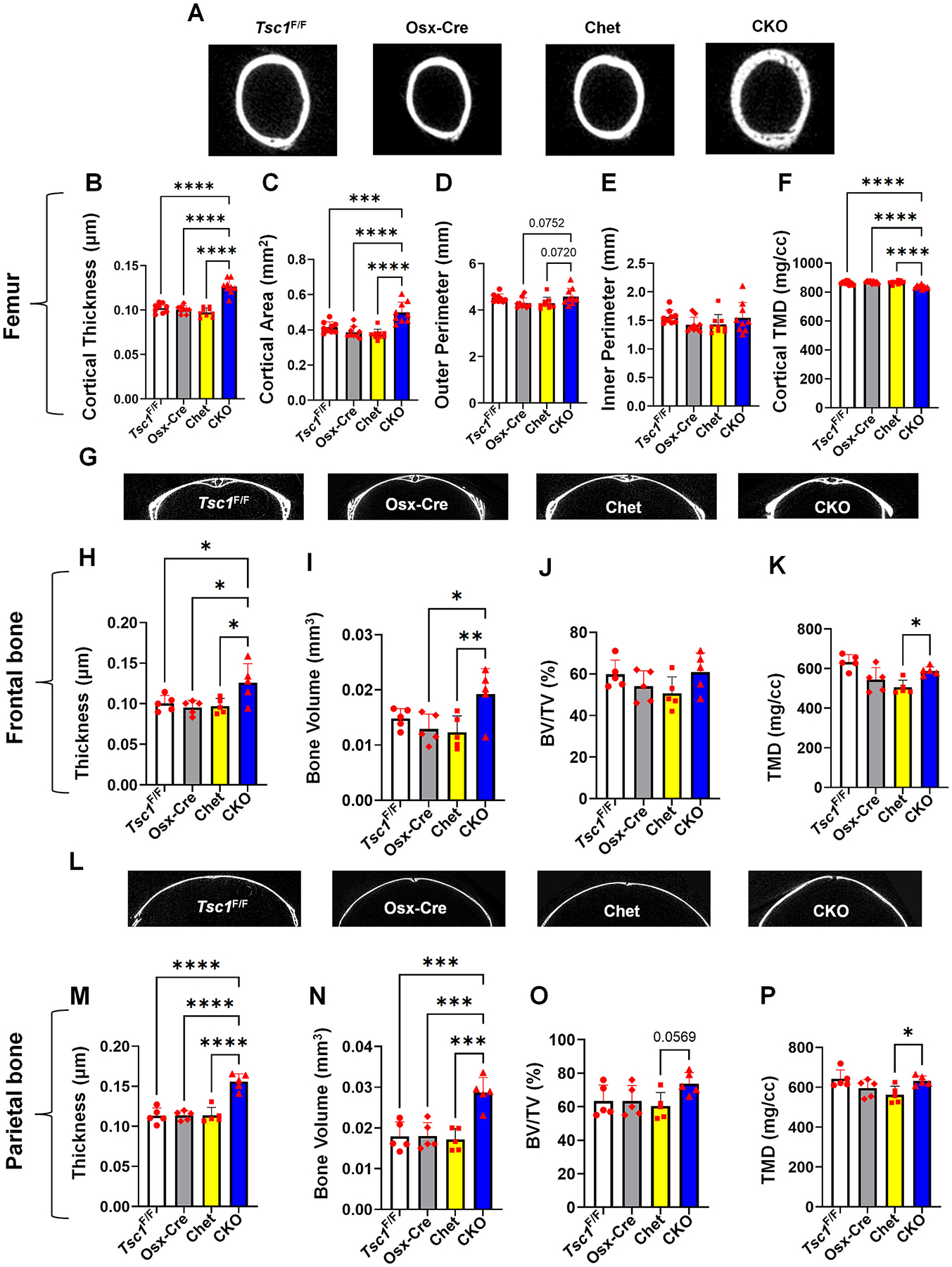
NanoCT analysis demonstrates higher femoral cortical and calvarial bone mass in one-month-old male mice lacking *Tsc1* in Osterix-expressing cells. (A-F) Representative nanoCT images (A) and quantification of femoral cortical bone parameters (B-F). CKO (*Tsc1*^F/F^;Osx-Cre) mice had significantly greater cortical thickness (B) and cortical area (C) than *Tsc1*^F/F^, Osx-Cre, and Chet controls. The increase was associated with a larger outer perimeter (C) without change in inner perimeter (E). Cortical TMD was modestly but significantly reduced in CKO mice (F). (G-K) Representative nanoCT images (G) and quantification of frontal bone parameters (H-K). CKO mice showed significantly greater thickness (H) and bone volume (I) compared with controls. BV/TV was not significantly altered (J). TMD was slightly but significantly higher in CKO mice than in Chet, with no difference from *Tsc1*^F/F^ or Osx-Cre controls (K). (L–P) Representative nanoCT images (L) and quantification of parietal bone parameters (M-P). CKO mice exhibited significantly greater thickness (M) and bone volume (N) compared with controls. BV/TV showed a trend toward increase compared with Chet only (*p* = 0.0569) (O). TMD was slightly but significantly higher in CKO mice than in Chet, with no difference from the other controls (P). Data are mean ± SD. *p < 0.05, **p < 0.01, ***p < 0.001, ****p < 0.0001 by one-way ANOVA with post hoc comparisons. Each dot represents one mouse; *n* = 6–8 per group depending on the measurement.

In the femur, CKO mice exhibited a 13–22 % greater cortical thickness compared with *Tsc1*^F/F^, Osx-Cre, and Chet mice (Fig. 1A, B). Cortical area was similarly 21–33 % greater (Fig. 1C). These changes were associated with a greater (*p* = 0.0720) outer perimeter (Fig. 1D) without a reduction in inner perimeter (Fig. 1E). Despite the greater cortical mass, femoral cortical bone in CKO mice showed a modest but statistically significant 3 % lower tissue mineral density (TMD) (Fig. 1F).

In the frontal bone, CKO mice had significantly greater thickness and bone volume compared with controls (Fig. 1G, H, I). However, bone volume fraction (BV/TV) was not significantly altered (Fig. 1J). TMD was slightly but significantly higher in CKO mice compared with Chet, but not compared with *Tsc1*^F/F^ or Osx-Cre controls (Fig. 1K).

In the parietal bone, CKO mice displayed significantly greater thickness and bone volume compared with controls (Fig. 1L, M, N). BV/TV showed only a trend toward higher values compared with Chet (*p* = 0.0569) and no significant difference from other controls (Fig. 1O). TMD was also slightly but significantly higher in CKO mice compared with Chet, with no significant difference from the other control groups (Fig. 1P).

Collectively, these findings demonstrate that *Tsc1* deletion in Osterix-expressing cells leads to a greater femoral cortical thickness and area, and calvarial bone mass, with limited effects on mineral density.

### 3.2. Tsc1-deficient cortical bone shows enhanced osteoblast proliferation in vivo in association with upregulation of osteoblast differentiation marker genes

**Fig. 2.**
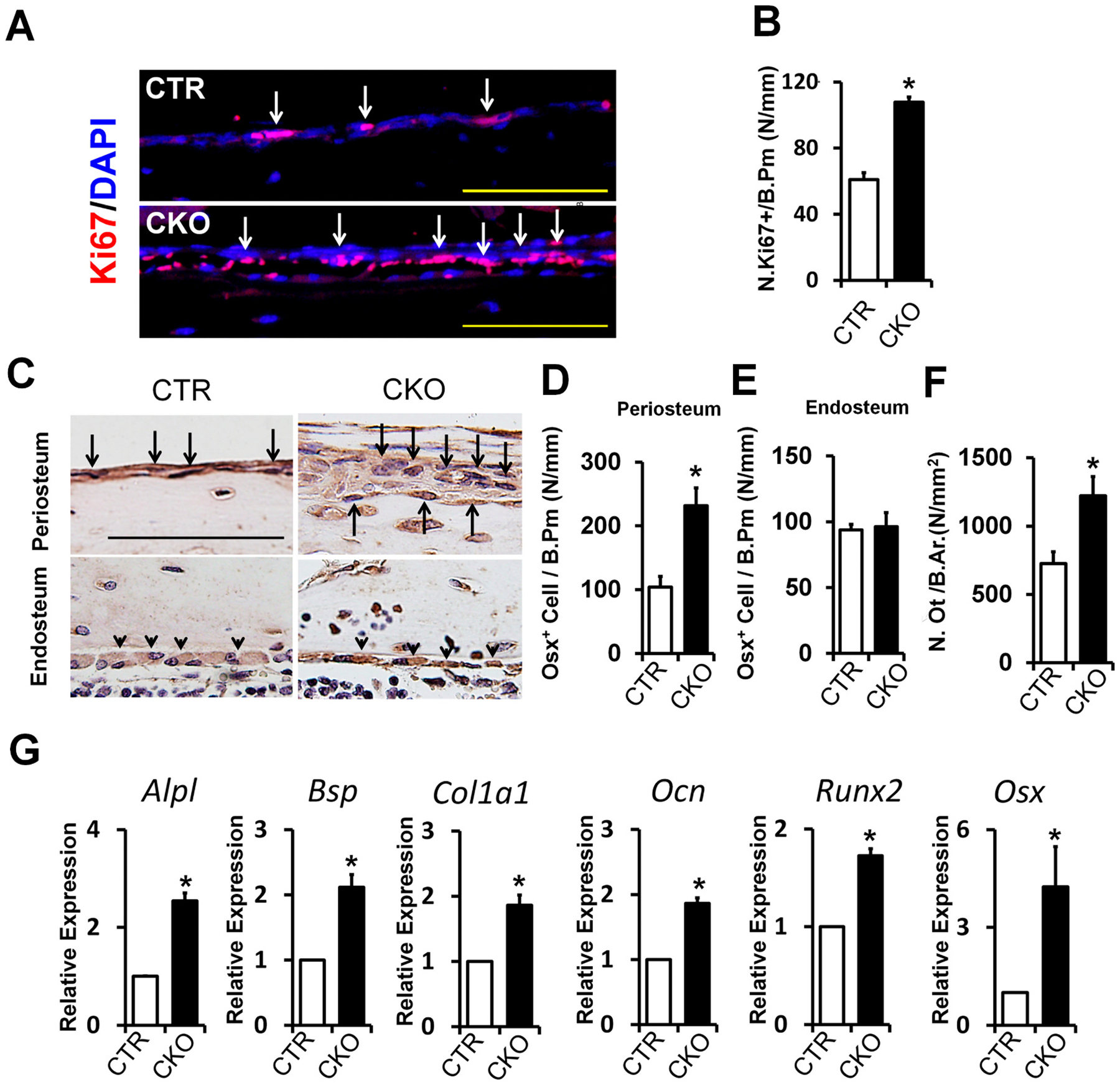
Enhanced osteoblast proliferation in cortical bone of one-month-old *Tsc1*-deficient mice. (A, B) Immunofluorescence staining with anti-Ki67 antibody in the femur of control (*Tsc1*^F/F^) and CKO (*Tsc1*^F/F^;Osx-Cre) mice. (A) Representative merged fluorescent images showing Ki67 (red) and DAPI (blue) in the periosteum. Scale bar = 50 μm. (B) Quantification of Ki67-positive cells per bone perimeter (B⋅Pm) (*n* = 4 per group). (C–E) Immunohistochemistry staining with anti-Osterix antibody in the femur of control (*Tsc1*^F/F^) and CKO (*Tsc1*^F/F^;Osx-Cre) mice. (C) Representative staining images showing the Osterix+ cells. The arrows point to osterix+ cell on the periosteal surface and the arrow heads point to osterix+ cell on the endosteal surface. (D-E) Quantification of Osterix-positive cells per bone perimeter in the periosteum (D) and endosteum (E) (*n* = 3 per group). Scale bar = 50 μm. (F) Quantification of osteocyte number per bone area (N.Ot/B⋅Ar). (G) Quantitative polymerase chain reaction (qPCR) analysis of mRNA expression of osteoblast differentiation markers (*Alpl*, *Bsp*, *Col1a1*, *Ocn*) and transcription factors (*Runx2*, *Osx*) in femoral cortical bone from control (*Tsc1*^F/F^) and CKO (*Tsc1*^F/F^;Osx-Cre) mice (n = 4 per group). Data in panels B, D, E, F, and G are presented as mean ± SE. *p < 0.05 by unpaired Student's *t*-test.

To explore the mechanisms underlying the cortical bone phenotype in *Tsc1*-deficient mice, we assessed osteoblast proliferation by quantifing Ki67-positive cells in the periosteum. CKO mice displayed 56 % more Ki67-positive cells compared with controls (Fig. 2A, B). Osterix-positive osteoblasts were more abundant in the CKO periosteum but not in the endosteum (Fig. 2C–E). Additionally, osteocyte number was significantly higher in CKO cortical bone (Fig. 2F).

Nex, we examined mRNA expression of osteoblast differentiation marker genes—alkaline phosphatase (*Alpl*), bone sialoprotein (*Bsp*), collagen type I alpha 1 (*Col1a1*), and osteocalcin (*Ocn*)—and osteoblast transcription factors—Runt-related transcription factor 2 (*Runx2*) and Osterix (*Osx/Sp7*). All six genes were significantly upregulated in femoral cortical bone from CKO mice compared with controls (Fig. 2G).

As reported previously, the majority of CKO mice died around one month of age for unknown reasons [11]. However, a very small number of survivors were available for analysis. NanoCT evaluation of femora from these two-month-old CKO mice revealed significantly higher trabecular and cortical bone mass (Fig. S1). Similar to the one-month-old CKO mice, the two-month-old CKO had increased cortical outer perimeter without changes in inner perimeter, suggesting the cortical bone gain occurred at the periosteal surface.

**Fig. 3.**
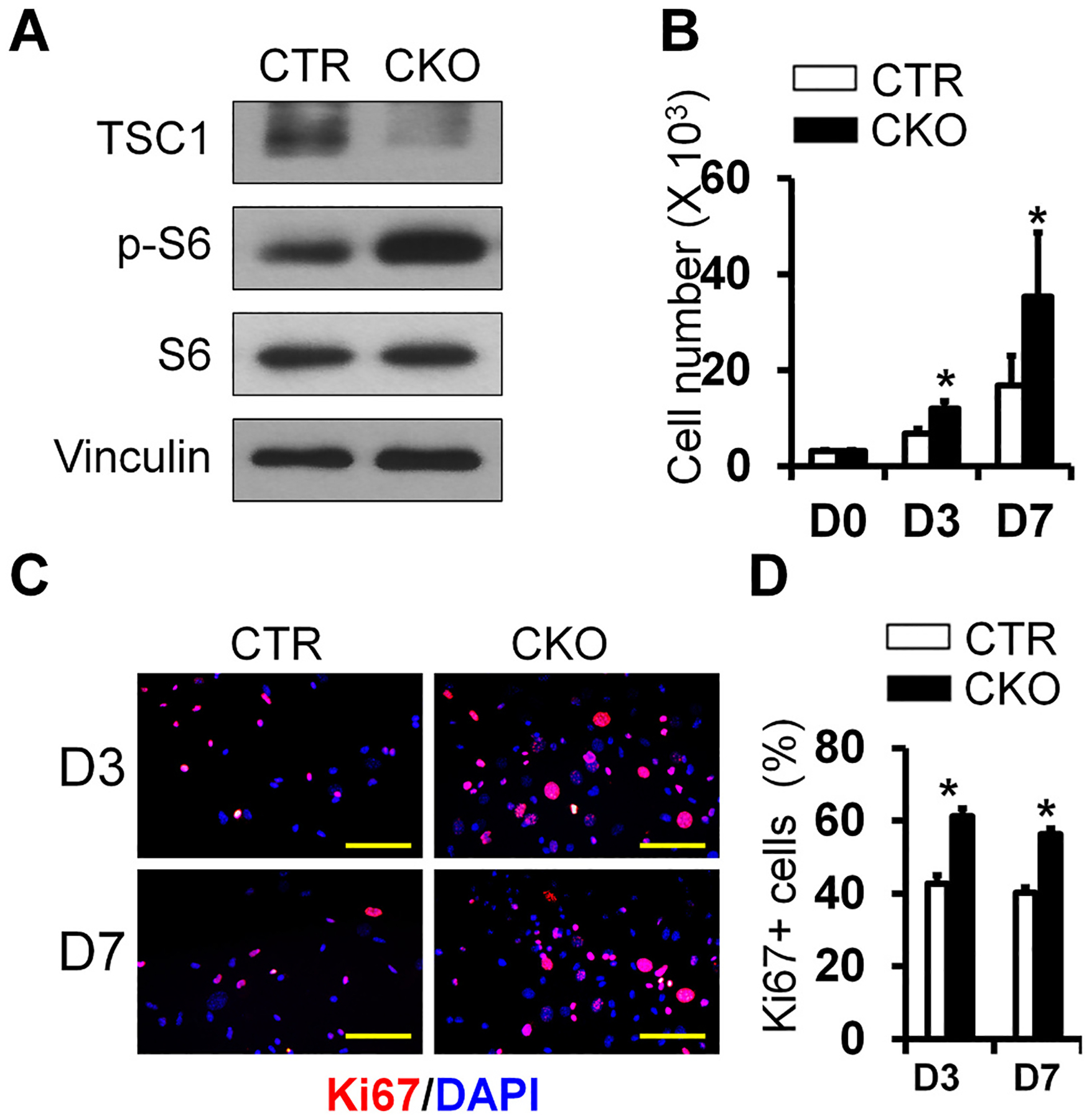
Enhanced proliferation of femur-derived osteoblasts lacking *Tsc1* in vitro. Osteoblasts were isolated from femurs of one-month-old mice as described in Materials and Methods. (A) Western blot analysis showing efficient deletion of TSC1 and increased levels of phosphorylated S6 (p-S6) in CKO (*Tsc1*^F/F^;Osx-Cre) osteoblasts compared with control (*Tsc1*^F/F^) cells; total S6 and vinculin served as loading controls. (B) Quantification of cell number after culture for 3 and 7 days in osteogenic medium (n = 3 per group). (C) Representative merged immunofluorescence images of Ki67 (red) and DAPI (blue) staining in control and CKO osteoblasts at day 3 and day 7 in the same culture system as in B. Scale bar = 50 μm. (D) Quantification of Ki67-positive cells in the cultures shown in C (n = 3 per group). Data are mean ± SE. *p < 0.05 by unpaired Student's *t*-test.

We further examined the effect of *Tsc1* deletion on femur-derived osteoblast proliferation in vitro. Western blot analysis confirmed efficient deletion of TSC1 in CKO osteoblasts, accompanied by elevated levels of phosphorylated S6 (p-S6), a marker of mTORC1 activation (Fig. 3A). TSC1-deficient osteoblasts exhibited enhanced proliferative capacity, as evidenced by a significant increase in cell number over time (Fig. 3B) and a higher percentage of Ki67-positive cells at both day 3 and day 7 compared with controls (Fig. 3C, D). However, in contrast to in vivo (Fig. 2), *Tsc1* deletion impaired osteogenic differentiation and mineralization in femoral osteoblast cultures in vitro (Fig. S2), consistent with our previous findings in *Tsc1*-deficient calvarial osteoblasts [7]. This discrepancy between in vivo and in vitro effects may reflect differences in the local environment, although the underlying mechanisms remain to be determined.

### 3.3. Postnatal Tsc1 deletion leads to higher bone mass and enhanced mechanical properties

**Fig. 4.**
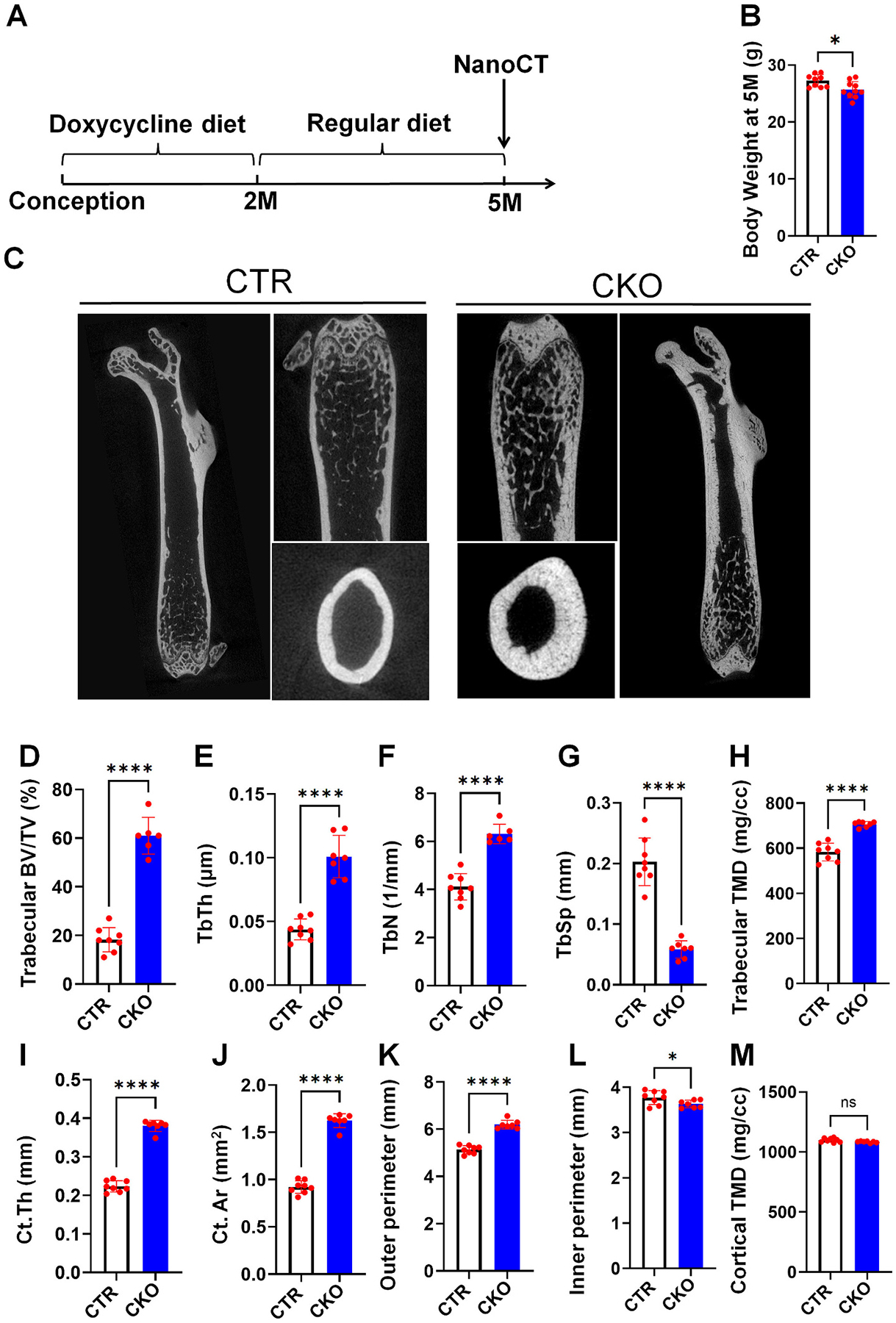
Postnatal *Tsc1* deletion leads to higher trabecular and cortical bone mass in adult male mice. (A) Experimental design: breeding pairs were maintained on a doxycycline (Dox)-containing diet, and offspring were indirectly exposed to Dox through maternal milk before weaning, followed by direct Dox feeding after weaning until 2 months of age. Mice were then switched to a regular diet for an additional 3 months before nanoCT analysis at 5 months of age. (B) Body weight at 5 months of age. (C) Representative nanoCT images of control (CTR, Tsc1F/F) and CKO (Tsc1F/F;Osx-Cre) mice. (D-H) Trabecular bone parameters: bone volume fraction (BV/TV), trabecular thickness (Tb.Th), trabecular number (Tb.N), trabecular separation (Tb.Sp), and trabecular tissue mineral density (TMD). (I–M) Cortical bone parameters: cortical thickness (Ct.Th), cortical area (Ct.Ar), outer perimeter, inner perimeter, and cortical TMD. Each dot represents one mouse. Data are mean ± SD. *p < 0.05, **p < 0.01, ***p < 0.001, ****p < 0.0001, ns=not significant by unpaired Student's t-test.

The early lethality of CKO mice prevented assessment of the effects of *Tsc1* deletion on bone at later postnatal stages [11]. To address this, we suppressed the Osx-Cre activity by feeding mice a Dox-containing diet [20,23]. Breeding pairs were maintained on a Dox-containing diet, and offspring were indirectly exposed to Dox through maternal milk until weaning, followed by direct Dox feeding after weaning until 2 months of age. At that point, mice were switched to a regular diet for an additional three months before bone analysis (Fig. 4A).

As an initial step, we examined femoral bone parameters at 2 months of age to determine the effectiveness of *Cre* suppression (Fig. S3A). This approach prevented early lethality, and CKO mice had body weights comparable to controls (Fig. S3B). Trabecular bone parameters were largely similar between CKO and control mice, except for a slight increase in trabecular number and a slight decrease in trabecular spacing in CKO mice (Figs. S3C–S3G). In contrast, CKO mice exhibited significantly increased cortical thickness, cortical area, and outer perimeter without changes in inner perimeter or tissue mineral density (TMD) (Figs. S3H–S3L), indicating incomplete suppression of *Osx-Cre* activity under the Dox diet.

We then assessed femoral bone parameters at 5 months of age, following three months of restored Osx-Cre activity. Although CKO mice had slightly lower body weight at this stage (Fig. 4B), they exhibited markedly higher bone mass (Fig. 4C). In trabecular compartment, there was higher bone volume fraction, thickness, number, and lower trabecular spacing, along with elevated trabecular TMD (Figs. 4D–4H). Most cortical bone parameters were also significantly higher, including cortical thickness, cortical area, and outer perimeter, with a reduction in inner perimeter, and no change in cortical TMD (Figs. 4I–4M). The reduction in inner perimeter suggested an endosteal bone gain besides the periosteal bone gain.

**Fig. 5.**
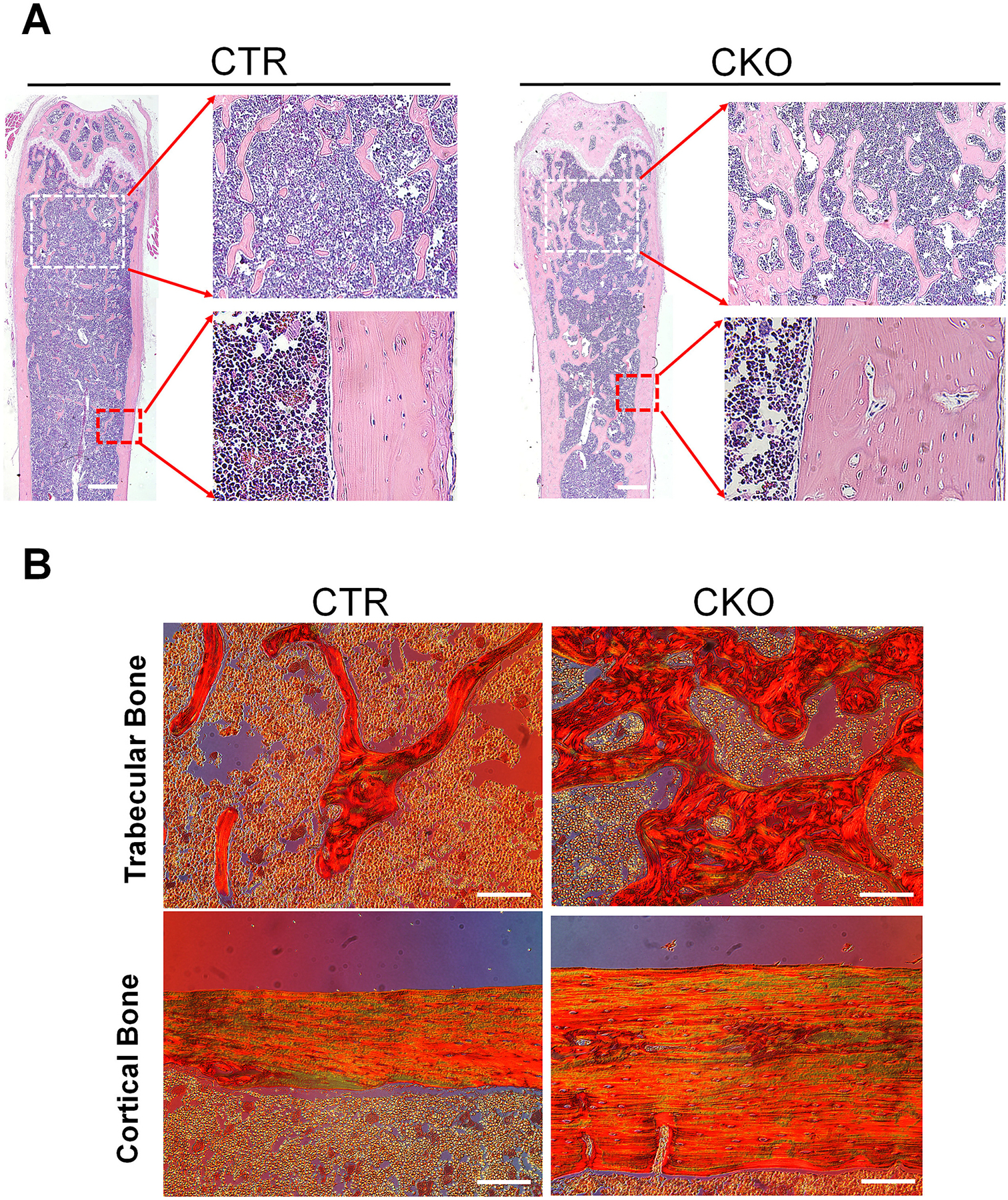
Histological and Picrosirius Red analyses of femoral bone. (A) Representative hematoxylin and eosin (H&E)–stained sections of femurs from control (CTR, Tsc1^F/F^) and CKO (*Tsc1*^F/F^;Osx-Cre) mice (the same cohort as in Fig. 4), showing greater trabecular and cortical bone mass in CKO femurs. (B) Picrosirius Red staining of femoral sections viewed under polarized light. Both control and CKO bones exhibited organized collagen with predominantly red birefringence and scattered green regions. The birefringence pattern in CKO mice was comparable to controls, indicating that the increased bone mass in CKO mice is composed of lamellar collagen with preserved organization. Scale bar = 1 mm for both panels.

Next, we performed histological assessment on the femoral bones. H&E staining showed that CKO mice had a robust bone gain in both trabecular and cortical compartments (Fig. 5A). In addition, Picrosirius Red staining of femoral sections was performed to assess collagen organization under polarized light (Fig. 5B). In control mice, the bone matrix exhibited bright red birefringent fibers with scattered regions of green signal, consistent with a mixture of mature and less mature collagen fibrils. In CKO mice, the birefringence pattern was comparable to that of controls, with well-organized red fibers and relatively fewer regions of green signal. These observations indicate that the additional bone formed in the CKO mice is composed of organized collagen with features suggestive of lamellar structure, supporting the interpretation that the increased bone mass is not associated with compromised collagen quality.

**Fig. 6.**
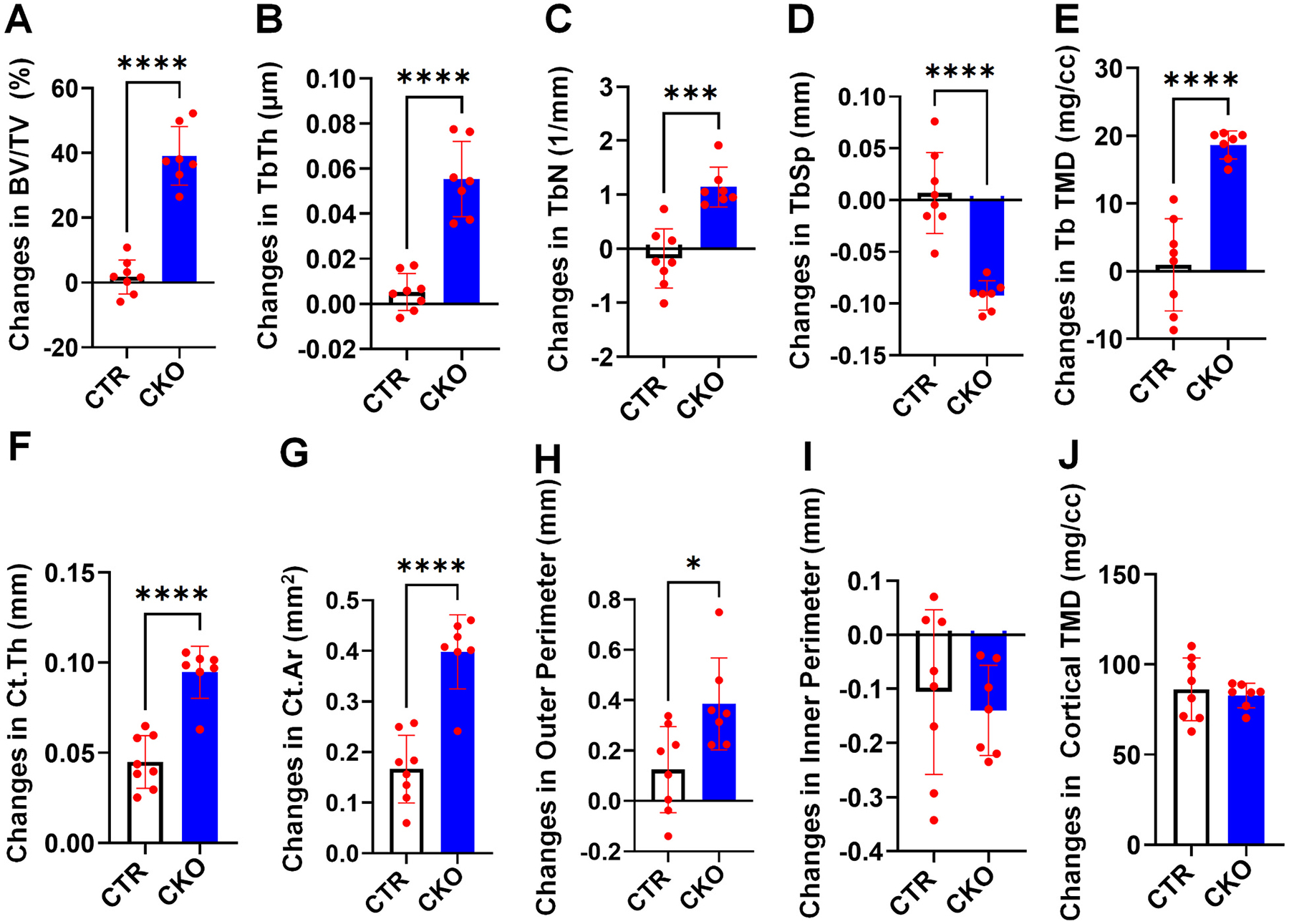
Bone gain following withdrawal of doxycycline diet is markedly enhanced in CKO mice. Changes in femoral bone parameters between 2 and 5 months of age in control (CTR, *Tsc1*^F/F^) and CKO (*Tsc1*^F/F^;Osx-Cre) mice were calculated as: values at 5 months (shown in Fig. 4) minus values at 2 months (shown in Fig. S3). (A–E) Trabecular bone parameters: change in BV/TV, Tb.Th, Tb.N, Tb.Sp, and TMD. (F–J) Cortical bone parameters: change in Ct.Th, Ct.Ar, outer perimeter, inner perimeter, and cortical TMD. Each dot represents one mouse. Data are mean ± SD. *p < 0.05, **p < 0.01, ***p < 0.001, ****p < 0.0001 by unpaired Student's *t*-test.

To better quantify bone gain following the restoration of Osx-Cre activity, we calculated the change in bone parameters between 2 and 5 months of age (Fig. 6). In the trabecular compartment, control mice showed minimal changes, whereas CKO mice had substantial increases in bone volume fraction, thickness, number, and TMD, along with decreased trabecular spacing (Figs. 6A–6E). In the cortical compartment, both control and CKO mice exhibited increases in thickness, area, and outer perimeter, but these gains were significantly greater in CKO mice (Figs. 6F–6H). Inner perimeter decreased in both groups, while cortical TMD increased, but these changes did not differ significantly between genotypes (Fig. 6I, J).

**Fig. 7.**
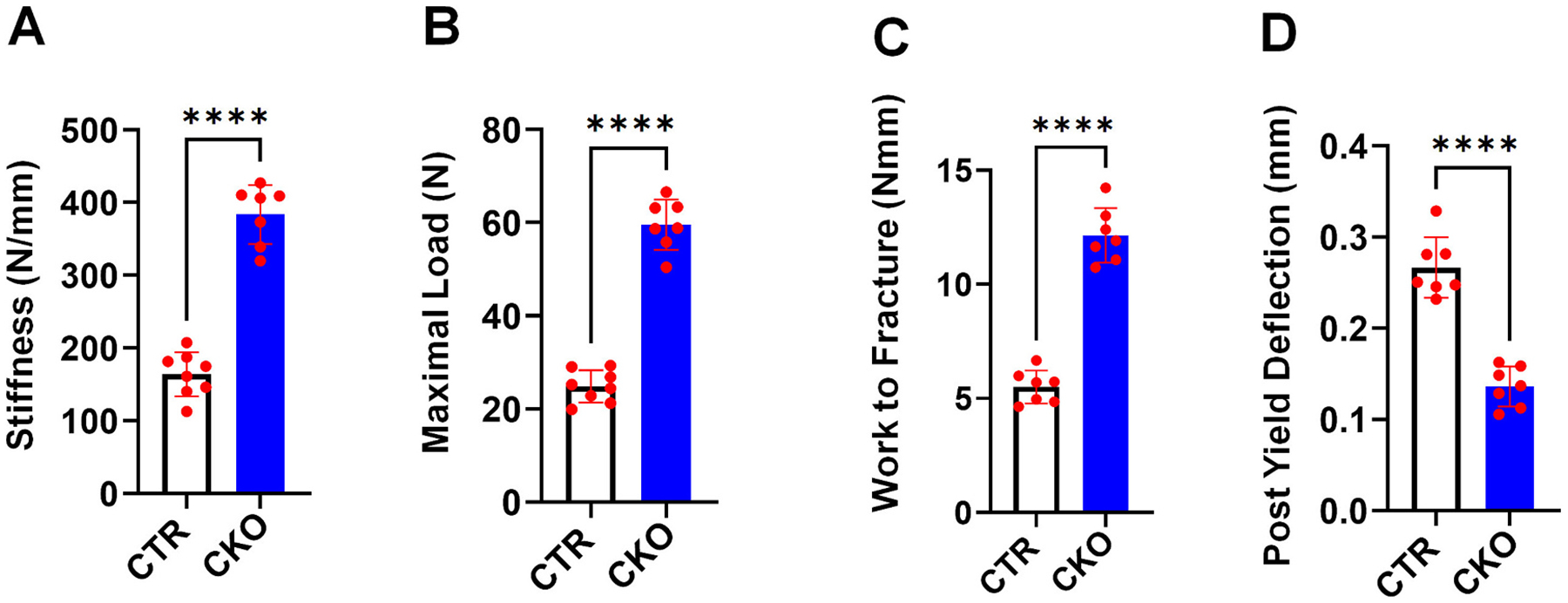
Postnatal *Tsc1* deletion improves femoral bone mechanical strength. Four-point bending tests were performed on femurs from control (CTR, *Tsc1*^F/F^) and CKO (*Tsc1*^F/F^;Osx-Cre) mice as described in Fig. 4. (A) Stiffness, (B) maximum load, (C) work to fracture, and (D) post-yield deflection. Each dot represents one mouse. Data are mean ± SD. ****p < 0.0001 by unpaired Student's t-test.

To evaluate the effect of *Tsc1* deletion on bone mechanical properties, we performed four-point bending tests on femurs. CKO mice exhibited significantly higher stiffness (Fig. 7A), maximum load (Fig. 7B), and work to fracture (Fig. 7C), along with reduced post-yield deflection (Fig. 7D), indicating enhanced mechanical strength.

**Fig. 8.**
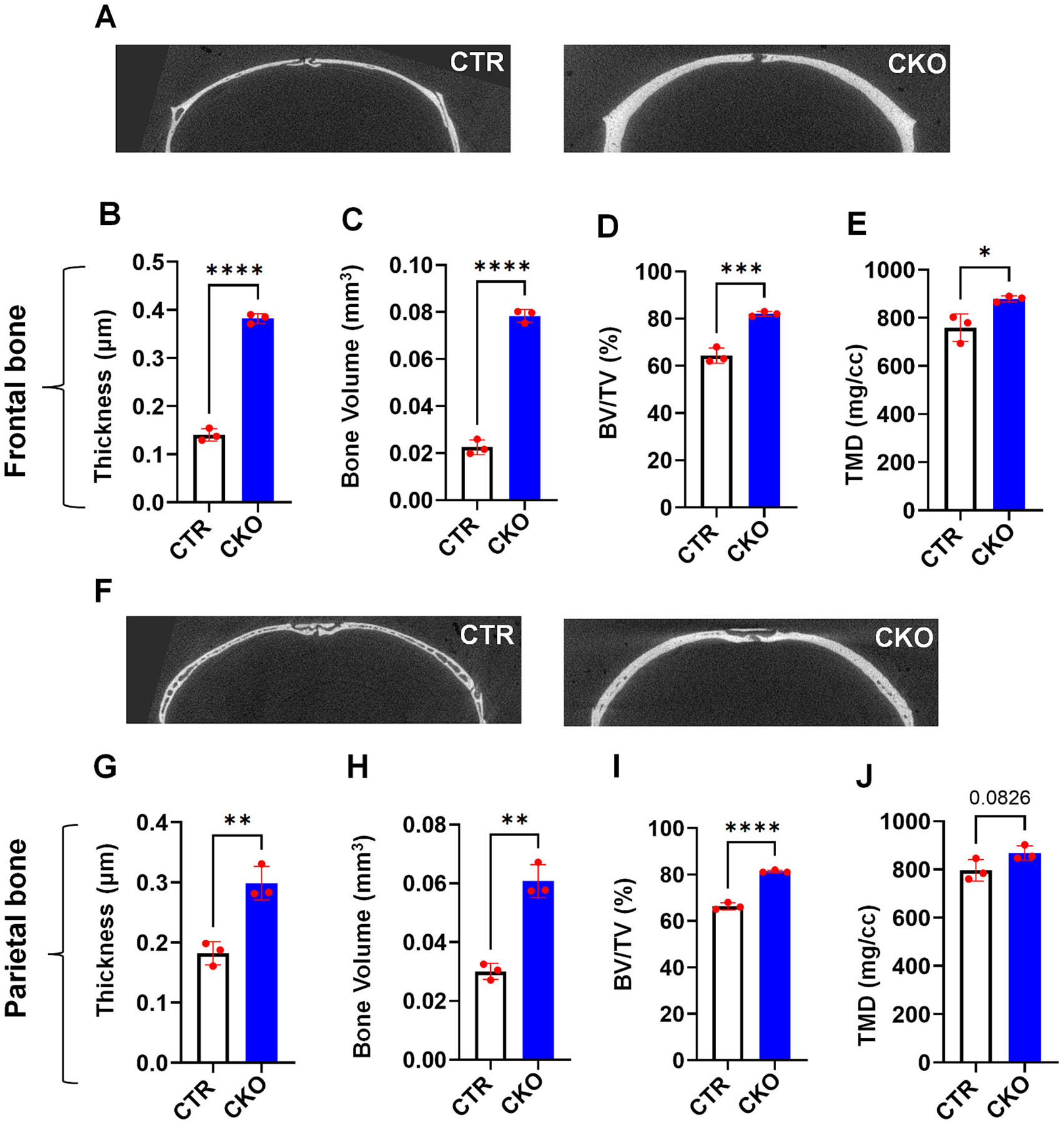
Postnatal *Tsc1* deletion leads to higher calvarial bone mass. Calvarial bone parameters measured by nanoCT in control (CTR, *Tsc1*^F/F^) and CKO (*Tsc1*^F/F^;Osx-Cre) mice as described in Fig. 4. (A-E) Representative nanoCT images (A) and quantification of frontal bone parameters (B-E). CKO mice have larger bone thickness (B), bone volume (C), bone volume fraction (BV/TV) (D), and tissue mineral density (TMD) (E). (F-J) Representative nanoCT images (F) and quantification of parietal bone parameters (G-J). CKO mice have higher bone thickness (G), bone volume (H), BV/TV (I), and TMD (J). Each dot represents one mouse. Data are mean ± SD. *p < 0.05, **p < 0.01, ***p < 0.001, ****p < 0.0001 by unpaired Student's t-test; *p* = 0.0826 indicated where applicable.

We next assessed the effect of *Tsc1* deletion on calvarial bone mass. In both the frontal (Figs. 8A–8E) and parietal bones (Figs. 8F–8J), CKO mice exhibited significantly greater thickness, bone volume, and bone volume fraction compared with controls. TMD was also significantly higher in the frontal bone of CKO mice, with a non-significant trend toward higher value in the parietal bone.

**Fig. 9.**
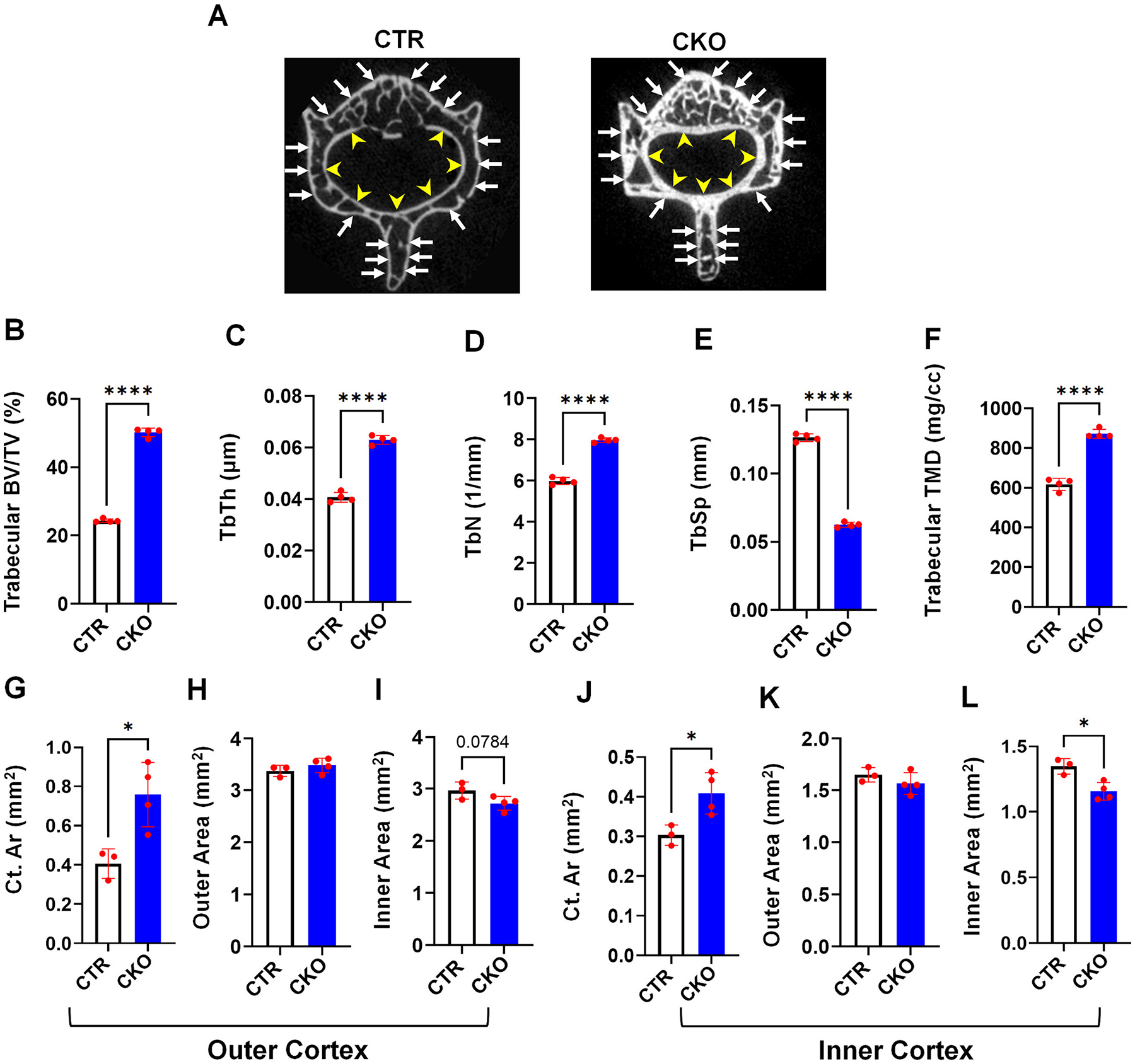
Post-natal *Tsc1* deletion leads to vertebral bone gain. NanoCT analysis of L3 vertebrae from control (CTR, *Tsc1*^F/F^) and CKO (*Tsc1*^F/F^;Osx-Cre) mice as described in Fig. 4. (A) Representative nanoCT images of LC3 vertebrae of CTR and CKO mice. Arrows point to outer cortex and arrow heads point to inner cortex. (B-F) Quantification of L3 vertebral trabecular bone parameters. CKO mice have higher bone volume fraction (BV/TV) (B), trabecular thickness (Tb.Th) (C), trabecular number (Tb.N) (D), trabecular separation (Tb.Sp) (E), and trabecular tissue mineral density (TMD) (F). (G-L) Quantification of L3 vertebral cortical bone parameters. CKO mice have higher cortical area (Ct.Ar) (G), no significant change in outer area (H), and a lower inner area (I) of outer cortex, and corresponding measures of the inner cortex (J–L). *p < 0.05, **p < 0.01, ***p < 0.001, ****p < 0.0001 by unpaired Student's t-test; *p* = 0.0784 indicated where applicable.

Lastly, we assessed the L3 vertebrae to determine whether postnatal *Tsc1* deletion also affected the axial skeleton. CKO mice showed robust bone gain (Fig. 9A). In the trabecular compartment, CKO mice exhibited significantly higher trabecular BV/TV, trabecular thickness, and trabecular number, along with lower trabecular spacing and higher trabecular TMD compared with controls (Figs. 9B–9F). In the cortical compartment (Figs. 9G–9L), CKO mice had significantly greater cortical area in both the outer cortex (Fig. 9G) and the inner cortex (Fig. 9J). Notably, the increase in cortical mass was driven primarily by bone gain on the inner cortical surface (Fig. 9I, L), whereas changes on the outer cortical surface were not significant (Fig. 9H, K).

## 4. Discussion

Our previous work demonstrated that deletion of *Tsc1* in Osx-expressing cells resulted in a significant reduction in trabecular bone mass at 1 month of age, accompanied by lower osteoblast numbers, higher osteoclast numbers, and robust bone marrow adiposity [11]. In that study, in vitro experiments using bone marrow stromal cells (BMSCs) revealed reduced proliferation and osteogenic differentiation along with enhanced adipogenic differentiation, mediated in part through suppression of Wnt/β-catenin signaling via an autophagy–Notch1 pathway [11]. In contrast, the present study found that the same *Tsc1* deletion led to a higher cortical bone mass in the femur at 1 month of age, and higher bone mass in both cortical and trabecular compartments at later time points. This apparent discrepancy suggests that the timing of analysis and the specific bone compartment examined can greatly influence the observed phenotype. The higher calvarial bone mass observed here is consistent with the cortical bone findings, potentially reflecting a shared bone formation mechanism. While calvarial bone is formed entirely through intramembranous ossification, later stages of cortical bone growth in the femur occur via appositional bone formation, which also relies on an intramembranous process, rather than the initial endochondral template formation.

The difference in trabecular bone mass between early and later stages may be explained by changes in osteoblast and osteoclast dynamics over time. At 1 month of age, we observed lower osteoblast numbers and higher osteoclast numbers in trabecular bone, which would favor bone loss [11]. However, in the current study, by 2 and 5 months of age, trabecular bone mass was significantly higher, indicating a shift toward net bone formation. Notably, Huang et al. using the same *Tsc1*^F/F^;Osx-Cre model reported lower osteoclast numbers and higher trabecular bone mass at 10 weeks of age [10]. Several similarities exist between that study and ours: (1) both show increased osteoblast proliferation in vivo—Huang et al. demonstrated this in femoral trabecular bone by BrdU labeling at 10 weeks, while we observed it in femoral cortical bone and in primary osteoblasts isolated from femoral cortical bone; (2) both report decreased in vitro osteogenic differentiation—our data using femur cortical bone-derived osteoblasts are consistent with our prior BMSC findings, while Huang et al. used calvarial osteoblasts; (3) both show increased cortical bone mass, with the expansion primarily at the periosteal surface, suggesting periosteal apposition as the main mechanism; and (4) both report increased trabecular bone mass at later stages. Differences between the studies may be partially attributable to genetic background, as our mice were on a C57BL/6 background and theirs on a 129S4/SvJae background, which could influence survival and skeletal phenotype.

A notable finding in this study is that *Tsc1* deletion improved bone mechanical properties, contrary to the prediction that mTORC1 hyperactivation yields bone with inferior mechanical properties. This view has been shaped by reports of disorganized bone in *Tsc1*- or *Tsc2*-deficient osteoblast-lineage models [9,10] and by work in the *Tsc1*^F/F^;Prx1-Cre model, where cortical bone mass increased but strength declined [8]. In the model of mTORC1 hyperactivation in osteoblasts since embryo stages [9,10], it is unknown whether the disorganization of bone translates into inferior mechanical strength because the higher bone mass may still lead to higher mechanical strength. Comparing to the model of mTORC1 hyperactivation in earlier mesenchymal lineage cells [8], the discrepancy with our results likely reflects both cell lineage and timing: Prx1-Cre acts in early mesenchymal progenitors, whereas Osx-Cre targets committed osteoblast-lineage cells. In addition, our post-natal model suppressed Osx-Cre activity until 2 months of age, avoiding chronic mTORC1 activation during early development and instead providing an anabolic stimulus later, potentially producing stronger bone. Importantly, Picrosirius Red staining under polarized light revealed that cortical bone in CKO mice exhibited well-organized collagen fibers with lamellar characteristics, comparable to controls. This indicates that the greater bone mass was not associated with disorganized or woven collagen, but instead maintained organized lamellar structure, consistent with the observed improvements in mechanical strength.

Differences in bone mineralization may also contribute to the divergent mechanical outcomes. Wu et al. did not report tissue mineral density (TMD) data, leaving open the possibility that reduced mineralization contributed to their observed deficits [8]. In our previous study targeting *Tsc1* in neural crest–derived cells [7]—developmentally related to mesenchymal progenitors at other skeletal sites—we observed markedly increased calvarial thickness and, in unpublished work, significantly elevated TMD, suggesting a potential enhancement of mechanical performance. In the present study, cortical TMD was unchanged, but trabecular TMD was significantly higher at 5 months—the age used for mechanical testing—indicating that *Tsc1* deletion does not inherently reduce mineralization. Although trabecular bone contributes little to whole-femur strength, these findings, together with prior work, suggest that mTORC1's effects on bone quality are context dependent, varying with lineage and timing of activation. They also raise the possibility that controlled, time-limited mTORC1 activation in adulthood could be leveraged to promote mechanically competent bone.

In summary, our results show that the skeletal impact of *Tsc1* deletion depends on bone compartment, developmental stage, and timing of mTORC1 activation. The improved mechanical properties in our post-natal model indicate that mTORC1 activation is not inevitably detrimental to bone quality and, if applied within a controlled time window, could potentially be harnessed to enhance bone mass and strength.

## Supplementary Material

1

## Data Availability

Data will be made available on request.

## Appendix A. Supplementary data

Supplementary data to this article can be found online at https://doi.org/10.1016/j.bone.2025.117695.
