# Supplementary material for: Loss of Tsc1 in Osterix-expressing cells leads to greater bone mass and strength in mice
Source: Bone. Author manuscript; Available in PMC 2026 Jun 19. (PMC13281960; doi:10.1016/j.bone.2025.117695)
Supplement: 1 [file NIHMS2185411-supplement-1.pdf]

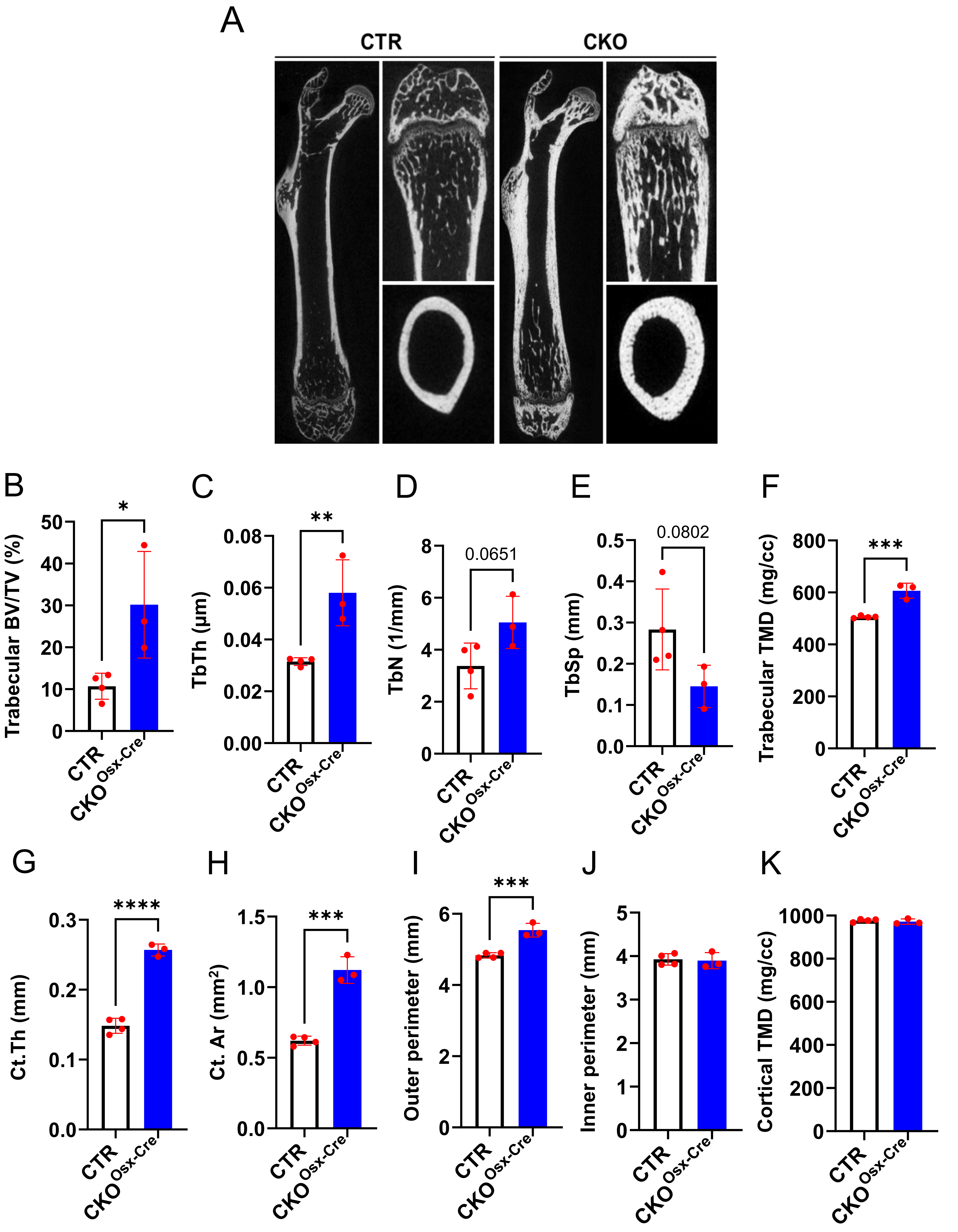

Figure S1. NanoCT analysis showing higher trabecular and cortical bone mass in two-month-old male mice lacking *Tsc1* in *Osx-Cre*-expressing cells.

Figure S1. NanoCT analysis showing higher trabecular and cortical bone mass in two-month-old male mice lacking *Tsc1* in *Osx-Cre*-expressing cells. (A) Representative longitudinal and transverse nanoCT images of distal femur from control (CTR, *Tsc1*$^{F/F}$) and CKO (*Tsc1*$^{F/F}$;Osx-Cre) mice. (B–F) Quantitative trabecular bone parameters: bone volume fraction (BV/TV) (B), trabecular thickness (Tb.Th) (C), trabecular number (Tb.N) (D), trabecular separation (Tb.Sp) (E), and trabecular tissue mineral density (TMD) (F). (G–K) Quantitative cortical bone parameters: cortical thickness (Ct.Th) (G), cortical area (Ct.Ar) (H), outer perimeter (I), inner perimeter (J), and cortical TMD (K). Each dot represents one mouse; n = 3-4 per group. Data are mean ± SD. *$p < 0.05$, **$p < 0.01$, ***$p < 0.001$, ****$p < 0.0001$ by unpaired Student's t-test.

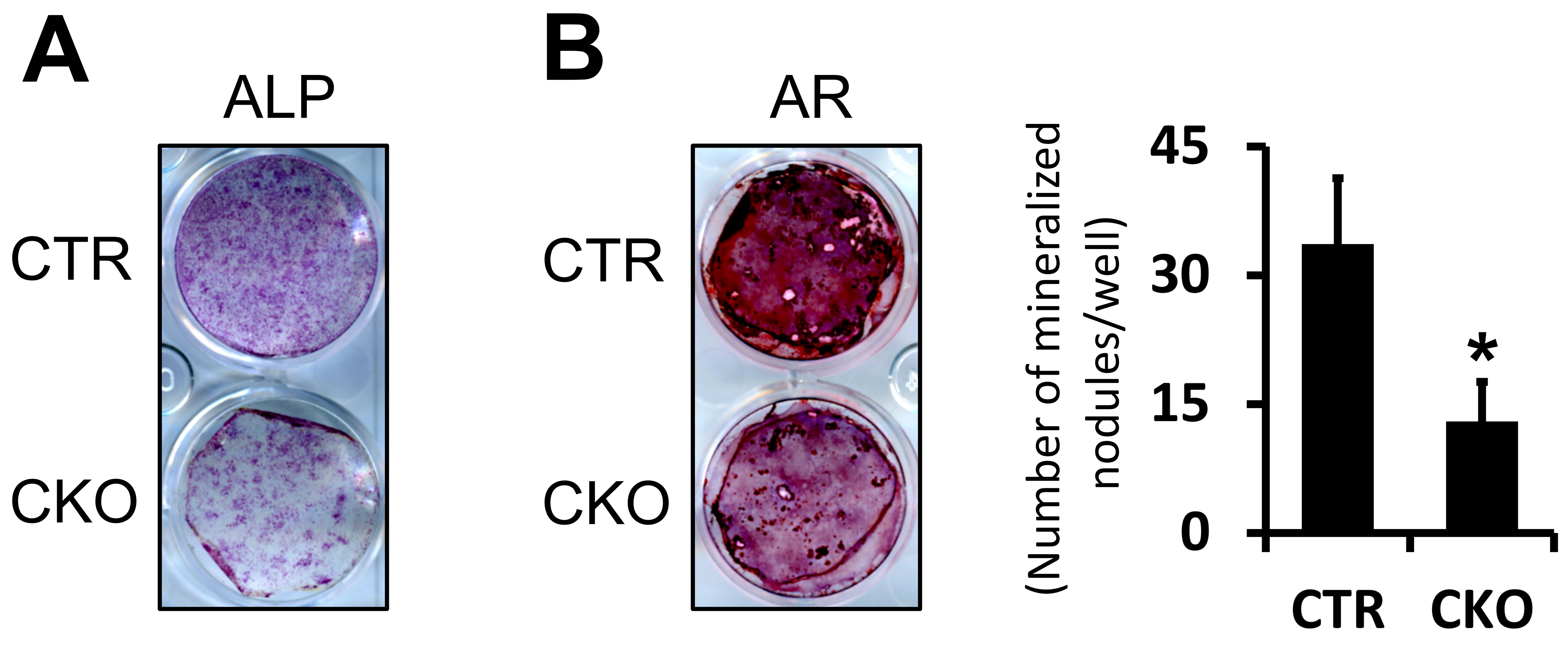

Figure S2. *Tsc1* deletion compromised osteogenic differentiation of long bone-derived osteoblasts in vitro. Osteoblasts were isolated from femur and cultured in osteogenic medium as described in Materials and Methods. Osteogenic differentiation and mineralization were analyzed by alkaline phosphatase (ALP) staining at day 7 (B) and alizarin red (AR) staining at day 21. Images shown were the representatives of three independent experiments.

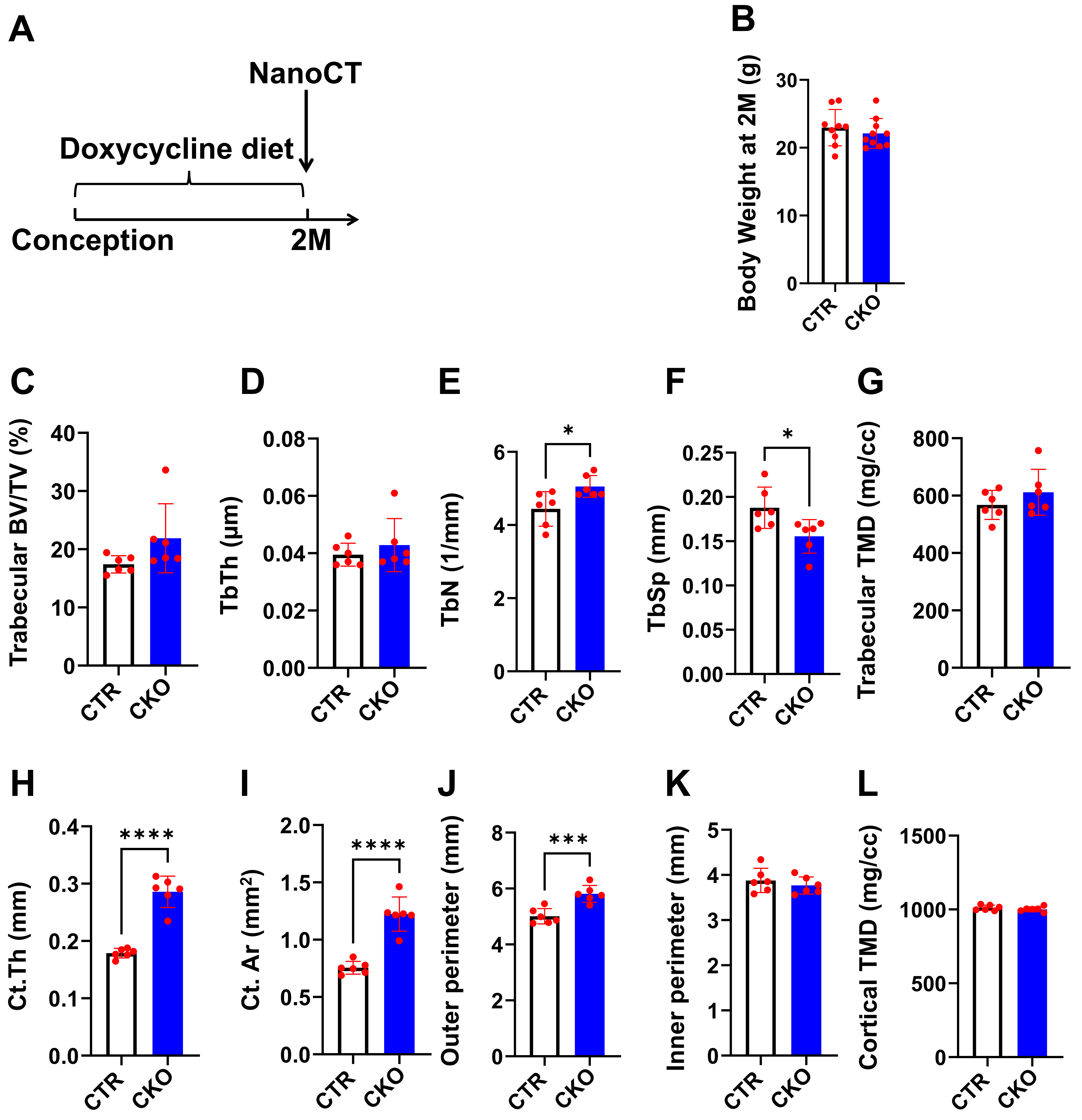

Figure S3. Doxycycline diet prevents early lethality but does not fully suppress *Osx-Cre* activity in CKO mice. (A) Experimental design: breeding pairs were maintained on a Dox-containing diet, and offspring were indirectly exposed to Dox through maternal milk before weaning, followed by direct Dox feeding after weaning until 2 months of age, when nanoCT analysis was performed. (B) Body weight at 2 months of age. (C–G) Trabecular bone parameters: BV/TV, Tb.Th, Tb.N, Tb.Sp, and trabecular TMD. (H–L) Cortical bone parameters: Ct.Th, Ct.Ar, outer perimeter, inner perimeter, and cortical TMD. Each dot represents one mouse; n = 6–8 per group. Data are mean ± SD. *$p < 0.05$, **$p < 0.01$, ***$p < 0.001$, ****$p < 0.0001$ by unpaired Student's t-test.
